# Technical success, clinical efficacy, and insight into the causes of restenosis after the percutaneous coronary intervention of *de novo* coronary artery lesions using a paclitaxel-coated balloon with citrate ester excipient

**DOI:** 10.3389/fcvm.2022.1012473

**Published:** 2022-10-31

**Authors:** Jerry Tervo, Jussi M. Kärkkäinen, Tuomas T. Rissanen

**Affiliations:** ^1^Heart Center, North Karelia Central Hospital, Joensuu, Finland; ^2^Heart Center, Kuopio University Hospital, Kuopio, Finland

**Keywords:** drug-coated balloon, drug-eluting balloon, bleeding risk, coronary artery disease, drug-coated balloon only

## Abstract

**Objectives:**

The aim of this all-comers registry study was to investigate the technical success, clinical efficacy, and safety of a drug-coated balloon (DCB) with paclitaxel combined with citrate ester excipient (CEE) in percutaneous coronary intervention (PCI) of *de novo* coronary lesions in an all-comers population.

**Materials and methods:**

A total of 338 consecutive PCIs using the DCB (CEE)-only approach comprising 406 *de novo* lesions were included in the study. Technical success was determined by the successful delivery of the device and no need for bailout stenting.

**Results:**

The mean follow-up time was 25 ± 12 months. The mean age of patients was 71 ± 11 years, and 48% had the acute coronary syndrome. A total of 55% of the patients were at risk of factor bleeding. The delivery of DCB was successful in 98% of cases. The overall technical success rate was 83%. Bailout stenting was used in 9% of lesions. Rotational atherectomy was used in 11% of cases before the DCB-only approach. The mean diameter of the DCBs used was 2.7 ± 0.5 mm and 38% of DCBs were large (≥3.0 mm). The 12-month MACE rates were 5.4 ± 1.7 and 18.3 ± 3.1% in stable CAD and in ACS, respectively. The respective target lesion revascularization (TLR) rates were 3.0 ± 1.3 and 8.5 ± 2.3%. Unacceptable acute recoil (>30%) was found in 74% of cases that needed repeat revascularization. No acute vessel closures occurred after DCB treatment.

**Conclusion:**

The DCB-only strategy using a paclitaxel-coated (CEE) device was technically feasible, safe, and effective in an all-comers population. Acute recoil was found as a significant cause of restenosis after the DCB-only strategy.

## Introduction

Percutaneous coronary intervention (PCI) using metallic drug-eluting stents (DES) is currently the mainstream approach in the percutaneous treatment of coronary artery disease (CAD). The concept of provisional stenting after achieving a stent-like result after predilatation was proposed already in the 1990s but this idea was limited by the high rate of restenosis after plain-old balloon angioplasty (1). Paclitaxel-coated balloons were initially developed for the treatment of in-stent restenosis (ISR) (2, 3). However, in the modern DES era, ISR is a relatively rare phenomenon comprising nowadays less than 5% of all indications for PCI. Despite increasing evidence on the efficacy of the drug-coated balloon (DCB)-only approach in *de novo* coronary artery lesions derived both from registry-based studies and randomized controlled trials (RCTs) (4–11), the ESC and AHA revascularization guidelines do not recognize the DCB-only approach in this indication at all (12, 13). Importantly, the paclitaxel-coated DCBs have been shown to be safe in the treatment of CAD in a large patient-level meta-analysis and a recent large RCT did not show any negative safety signal in the use of DCBs for the treatment of peripheral arterial disease (14, 15).

The DCB-only approach has potential in some anatomical and clinical scenarios where permanent implantation of a metallic coronary implant may lead to suboptimal clinical outcomes. In small coronary vessels, DCB was found to be non-inferior to the implantation of DES in an RCT (7). In long and diffuse lesions, a “full metal jacket” often compromises side branches and may lead to a less favorable long-term outcome. Prolonged dual-antiplatelet therapy (DAPT) may be harmful to elderly, high-bleeding risk (HBR) patients, e.g., those on oral anticoagulation (OAC) (16). The DEBUT RCT showed that the treatment of HBR patients with the DCB-only strategy combined with short DAPT is safe and efficacious (8).

In this study, we investigated the clinical efficacy of a recently developed DCB that has a coating of citrate ester excipient (CEE) and a smaller concentration of paclitaxel (2 μg/mm^2^) than the majority of other DCBs. Previously, this DCB has been shown to be effective for the treatment of ISR in an RCT with similar late-lumen loss to paclitaxel-coated DCB with iopromide as an excipient (3). We studied the efficacy of this DCB in *de novo* coronary artery lesions in a retrospective all-comers population with special emphasis on the technical success of the DCB-only approach. The subgroups of the study were small and large vessels as well as patients with stable CAD and ACS (acute coronary syndromes).

## Materials and methods

### Study population

This is a retrospective, single-center, all-comers registry study of consecutive patients undergoing DCB-only PCI between August 2014 and November 2018 for *de novo* lesions using a drug-coated balloon (DCB) with citrate ester excipient (CEE), i.e., acetyl tributyl citrate, 2 μg/mm^2^ (Agent, Boston Scientific). The inclusion criterion for the study was that at least one *de novo* lesion was treated with the DCB-only approach using this DCB. The decision on the DCB-only strategy was done at the operator’s discretion after the predilatation of the lesion. Patients presenting either with stable CAD or ACS (unstable angina, non-ST elevation, and ST elevation) were included. The only exclusion criterion in this study was ISR. In 338 PCI sessions, a total of 406 *de novo* lesions were treated with the DCB-only approach.

The patient characteristics are shown in Table 1. The mean age of the patients was 71 ± 11 years. However, 24% of patients were older than 80 years. A total of 37% of the patients were diabetics and 22% had had a prior myocardial infarction. HBR is an important clinical indication for the DCB-only approach (8). In this cohort, 55% of the patients were considered to be at HBR, e.g., due to advanced age or use of OAC (21% of patients). A total of 48% of patients presenting with ACS and as many as 25% had STEMI at the index procedure.

**TABLE 1:** Baseline characteristics of patients including risk factors, comorbidities, bleeding risk, acute coronary syndrome (ACS) and Canadian cardiovascular society (CCS) class as well as ejection fraction.

	All	%	Stable CAD	%	ACS	%
	*n*	%	*n*	%	*n*	%
**Demographics and comorbidities**	338		177		161	
Age, years	71 ± 11		69 ± 11		73 ± 12	
Sex, male	230	68	118	67	112	70
Smoker	49	14	27	15	22	14
Ex-smoker	70	21	36	20	34	21
Diabetes	126	37	64	36	62	39
Hypertension	238	70	133	75	105	65
Prior myocardial infarction	73	22	38	21	35	22
Hypercholesterolemia (chol. > 5 or LDL > 2,5 mmol/L) or statin	260	77	148	84	112	70
**Bleeding risk factors, at least one, %**						
Anticoagulation	72	21	38	21	34	21
Anemia	115	34	50	28	65	40
Age ≥80 years	82	24	30	17	52	32
Active malignant disease	5	1	2	1	3	2
Prior stroke	30	9	11	6	19	12
Severe renal dysfunction (eGFR < 30 mL/kg/min)	5	1	2	1	3	2
Severe liver dysfunction (Bil > 2x or ALAT > 3x)	0	0	0	0	0	0
Planned elective surgery < 12 months after PCI	15	4	11	6	4	2
General frailty or cachexy (BMI < 20 kg/m2)	5	1	1	1	4	2
Patient not compliant to use DAPT on a regular basis	12	4	6	3	6	4
Prior bleeding requiring intervention^a^	21	6	11	6	10	6
**ACS class**						
Unstable angina or NSTEMI	121	36	–	–	121	75
STEMI	40	12	–	–	40	25
**CCS class^b^**						
CCS1	17	5	16	9	1	1
CCS2	120	36	100	56	20	12
CCS3	118	35	56	32	62	39
CCS4	75	22	3	2	72	45
**Ejection fraction (%)^c^**						
<30%	7	2	4	2	3	2
30–49%	80	24	32	18	48	30
≥50%	131	39	65	37	66	41

^a^Medical or surgical, ^b^Data available on 330 patients, ^c^Data available on 218 patients. ACS, acute coronary syndrome; CCS, Canadian cardiovascular society.

### Drug-coated balloon-only percutaneous coronary intervention

The majority of patients (96%) were on aspirin (100 mg daily) beforehand or received a loading dose of aspirin 250–500 mg i.v. or p.o. just before PCI. In stable CAD, patients received a loading dose of clopidogrel (600 mg) followed by 75 mg per day before or immediately after PCI. Patients presenting with ACS were mainly treated with ticagrelor (63%). A total of 25% and 6% of the patients were prescribed clopidogrel and prasugrel, respectively. The mean duration of DAPT was 3.8 ± 3.6 months in stable CAD and 7.2 ± 6.7 months after ACS. The recommendation of the manufacturer of Agent DCB (Boston Scientific) is 3 months in stable CAD. The duration of DAPT was at the operator’s discretion. A total of 13% and 15% of patients with stable CAD and ACS, respectively, were discharged with single antiplatelet therapy (SAPT). These patients were considered to be at very high HBR because of recent severe bleeding, malignancy, or urgent upcoming non-cardiac surgery. In 4.4% of cases, ADP receptor blocker was not used at all during or after PCI.

The DCB-only PCI was performed according to the international consensus group guidelines (17). The predilatation of the target lesion was mandatory before the application of DCB using the reference vessel-to-balloon diameter ratio of 1:1. Rotational atherectomy was used in 11% of cases for debulking of calcium. The DCB was dilated at least for 30 s to allow drug transfer to the vessel wall. Bailout stenting was done at the operator’s discretion, usually in case of flow-limiting dissection (TIMI < 3) or significant recoil (>30% in large vessels), after the application of DCB. In STEMI, thrombus aspiration was performed before DCB-only PCI in case of visible thrombus in the lesion.

### Endpoints and statistical analysis

The postoperative care of the patients and the follow-up were done according to normal local practices. The primary endpoints were major adverse cardiac events (MACE) and ischemia-driven target lesion revascularization (TLR) at 12 and 24 months. MACE was defined as cardiovascular death, non-fatal myocardial infarction, or TLR. Secondary endpoints were total and cardiovascular mortality. Bleeding episodes were analyzed according to the Bleeding Academic Research Consortium (BARC) criteria (18). Technical success of DCB-only PCI was defined as the absence of delivery failure of DCB or bailout stenting after DCB treatment due to the flow-limiting dissection (<TIMI 3) or significant recoil (>30%). Clinical endpoints were derived from the medical record system used by all the healthcare providers in the catchment area (Mediatri, Mediconsult, Finland). Data on the causes of death were obtained from the population registry of Finland. Cumulative MACE, TLR, and mortality rates were estimated using the Kaplan–Meier method, and the log-rank test was utilized for comparison between the groups (stable CAD, ACS, and large and small vessels). Angiographical evaluation of all TLR events at 12 months (19 events) was done to understand the underlying mechanism of TLR. Flow-limiting dissection and recoil >30% was considered a technical failure after DCB-only PCI as indicated by the consensus document (17). All statistical analyses were performed using the SPSS version 24.0 (IBM Corp., Armonk, NY, USA). The trial was approved by the Research Ethics Committee of the Northern Savo Hospital District.

## Results

### Technical success rate of the drug-coated balloon-only strategy

A total of 338 consecutive PCIs using the DCB-only approach comprising 406 lesions were performed. All lesions were *de novo* either in native coronary arteries or in vein grafts (Table 2). The most common target vessel was the left anterior descending artery (33%) followed by marginal and diagonal branches (25%) and the right coronary artery (22%). Two or more lesions were treated in 64% of the cases. The mean number DCBs used per lesion was 1.2. The sizes of predilatation balloons and DCBs used are presented in Table 2. The mean diameter of DCBs was 2.7 ± 0.5 mm while 38% of DCBs were large, i.e., ≥3.0 mm. Mostly used diameters of DCBs were 2.5–3.0 mm (70%) and mostly used lengths were 15–20 mm (70%). The lesions that were not treated by the DCB (CEE) stategy, received treatment by other paclitaxel-DCB, DES, a bare-metal stent (BMS), or POBA at the operator’s dicretion (Table 2).

**TABLE 2:** Baseline characteristics of percutaneous coronary intervention (PCIs) and devices.

	*n*	%
PCIs	338	100
Lesions treated with PCI	686	100
**Number of lesions treated with**		
Any DCB	497	72
DCB (CEE)	406	59
DES	182	27
BMS	2	0
POBA	5	1
**Number of lesions treated per patient**		
1 lesion	121	36
2 lesions	124	37
3 lesions	60	18
4 lesions	28	8
5 lesions	5	1
Rotational atherectomy + DCB (CEE)	44	11
**Target vessel of DCB (CEE)**		
LM	9	2
LAD	134	33
RCA	90	22
LCX	52	13
Marginal or diagonal branch	101	25
RPD or RPL	13	3
Vein graft	7	2
**Largest predilatation balloon diameter (mm)^a^**		
2.0	45	15
2.5	136	45
2.75	4	1
3.0	84	28
3.25	1	0
3.5	25	8
4.0	9	3
**DCB (CEE) diameter (mm)^b^**		
2.0	54	13
2.25	34	8
2.5	138	34
2.75	28	7
3.0	105	26
3.5	35	9
4.0	11	3
**DCB (CEE) length (mm)^c^**		
12	49	12
15	136	34
20	146	36
30	69	17
n.a.	5	1

BMS, bare metal stent; DCB, drug-coated balloon; CEE, citrate-ester excipient; DES, drug eluting stent; LAD, left anterior descending artery; LCX, left circumflex artery; POBA, plain old balloon angioplasty; RCA, right coronary artery; RPD, right posterior descending artery; RPL, right posterior lateral artery.

^a^Data available on 304 lesions treated with Agent DCB.

^b^Data available on 405 Agent DCBs.

^c^Data available on 405 Agent DCBs. n.a., Data not available.

The overall technical success of DCB-only PCI was 83% (Table 3). Delivery failure of DCB occurred only in nine cases (2%). In the *post hoc* analysis of the angiograms, the need for bailout stenting was found in 15% of cases after the DCB-only approach according to the criteria of the consensus document (<TIMI3 flow due to dissections or >30% recoil) (17). However, bailout stenting was eventually done only in 9% of cases. In 25 cases, bailout stenting was omitted and the suboptimal angiographic result was accepted due to contraindications for DAPT because of extreme HBR, such as recent life-threatening bleeding or upcoming urgent non-cardiac surgery. The reasons for bailout stenting were flow-limiting dissection (61%) and recoil (≥30%, 39%). The majority (93%) of bailout stents were DES. In three cases the bailout stent could not be delivered to the lesion due to anatomical complexity. There were no acute vessel closures in the 338 DCB (CEE)-only PCIs.

**TABLE 3:** Reason for technical failure of drug-coated balloon (DCB)-only percutaneous coronary intervention.

	*n*	%
Number of lesions attempted to be treated with DCB	415	100
Delivery failure of DCB	9	2
Successful delivery of DCB	406	98
Technical success of DCB-only PCI*	343	83
Number of lesions with indication for BOS	63	15
Stented due to flow-limiting dissection	19	30
Stented due to acute recoil ≥30%	16	25
Delivery failure of BOS	3	5
Dissection or recoil accepted without BOS	25	40
Number of BOSs used	42	100
DES	39	93
BMS	3	7

DCB, Drug-coated balloon; BOS, bailout stenting. TLR, target lesion revascularization.

*Defined by successful delivery and no need for BOS after DCB-only PCI.

### Clinical endpoints

**FIGURE 1:**
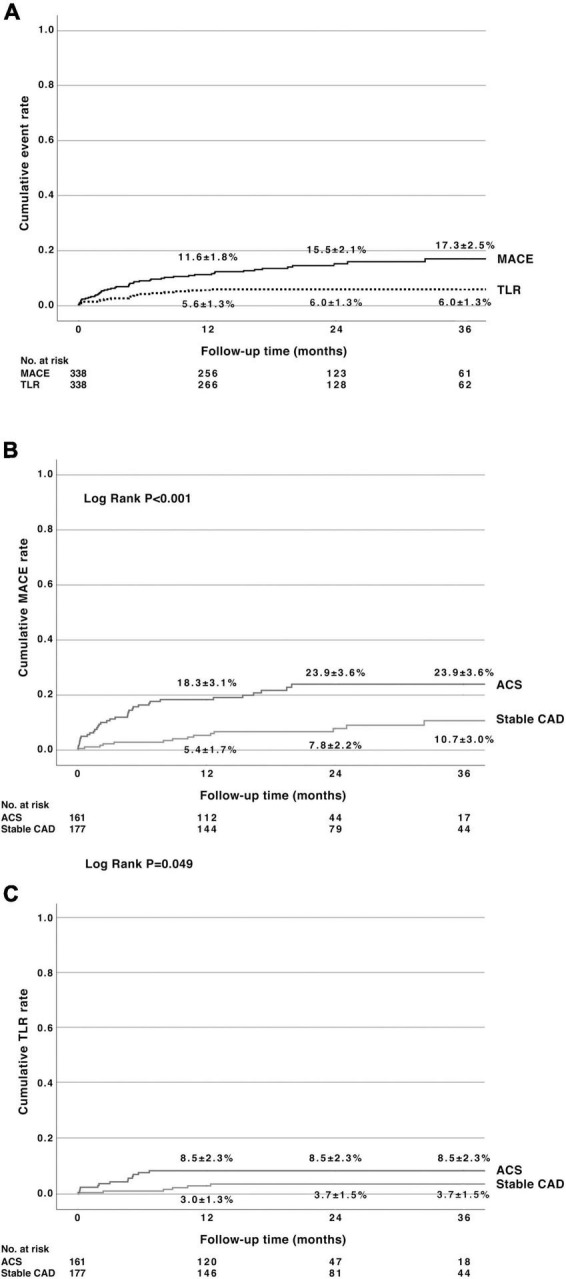
**(A)** Cumulative major adverse cardiac events (MACE) rate [consisting of cardiac death, non-fatal myocardial infarction, and target lesion revascularization (TLR)] and cumulative TLR rate after percutaneous coronary intervention using paclitaxel drug-coated balloon with citrate ester excipient as an excipient. **(B)** Cumulative MACE rate in patients with stable coronary artery disease (CAD) or having acute coronary syndromes (ACS). **(C)** Cumulative TLR rate in stable CAD and having ACS. MACE, major adverse cardiovascular events; TLR, target lesion revascularization; CAD, coronary artery disease; ACS, acute coronary syndromes.

**FIGURE 2:**
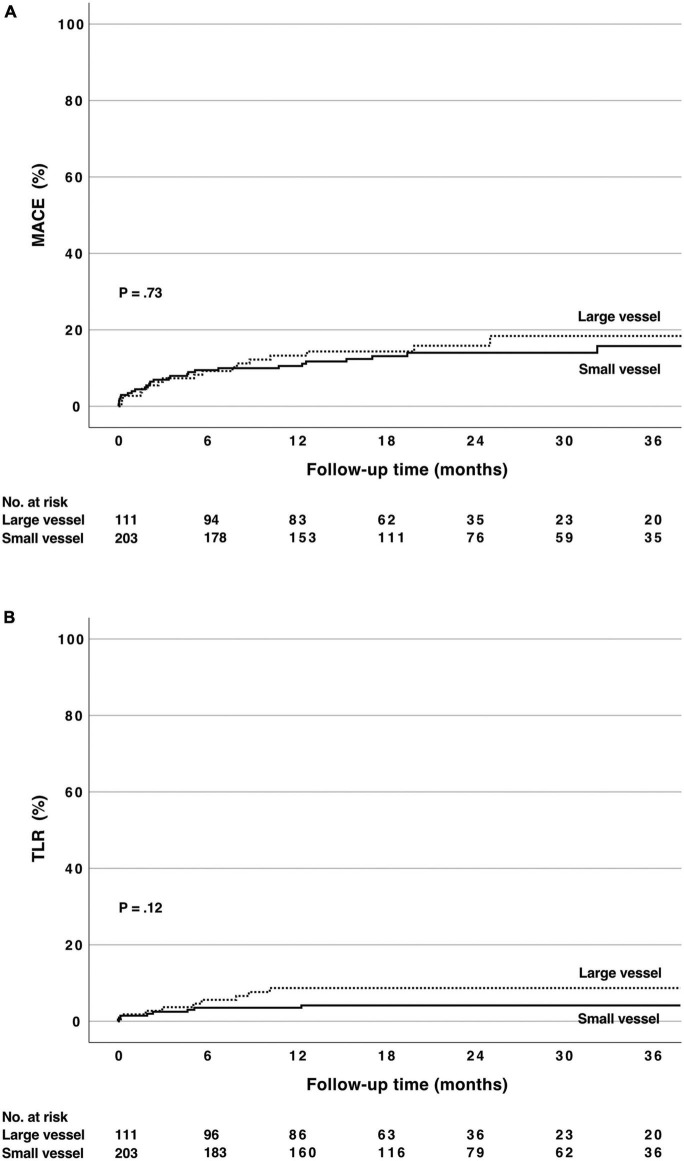
**(A)** Cumulative major adverse cardiovascular events rate [consisting of cardiac death, non-fatal myocardial infarction and target lesion revascularization (TLR)] after PCI using paclitaxel drug-coated balloon with citrate ester excipient as an excipient in small (≤2.75 mm) and large vessels (>2.75 mm). **(B)** Cumulative TLR rate in the same subgroups. There was no statistically significant difference between the groups (log rank p = ns). MACE, major adverse cardiovascular events; TLR, target lesion revascularization; DCB, drug-coated balloon; CEE, citrate ester excipient.

The median follow-up time was 23 months (interquartile range 16–36 months) for survival and 19 months for MACE (interquartile range 19–31 months). In this all-comers population consisting of elderly patients presenting both with stable CAD and ACS (25% presenting with STEMI), total mortality after DCB-only PCI was 2.3 and 12.6% at 12 months in stable CAD and ACS, respectively (*p* = 0.17; Table 4). Cardiovascular death occurred in 1.1% of patients with stable CAD and 8.8% in the ACS group at 12 months (*p* = 0.004). The respective MACE rates were 5.4 ± 1.7 and 18.3 ± 3.1% (*p* < 0.001). The TLR rate was 3.0 ± 1.3% in stable CAD and 8.5 ± 2.3% in patients presenting with ACS at 12 months (*p* = 0.049). Figure 1 shows the Kaplan–Meier estimates of MACE and TLR. The MACE and TLR rates did not differ significantly between small and large vessel groups (Table 4 and Figure 2). Significant bleeding (BARC 2-5) occurred in 33% of patients during the follow-time. At 12 months, BARC 2-5 bleeding rate was 24 and 32% in patients with stable CAD and ACS, respectively.

**TABLE 4:** The primary endpoints of the study at 12 and 24 months.

Timepoint (months)	12	24	*p* *
**Total mortality (%)**	7.2 ± 1.4	10.6 ± 1.8	
Stable CAD	2.3 ± 1.1	7.3 ± 2.1	0.017
ACS	12.6 ± 2.6	14.2 ± 2.8	
**Cardiovascular death (%)**	4.8 ± 1.2	5.6 ± 1.3	
Stable CAD	1.1 ± 0.8	1.8 ± 1.0	0.004
ACS	8.8 ± 2.2	9.7 ± 2.4	
**MACE rate (%)**	11.6 ± 1.8	15.5 ± 1.8	
Stable CAD	5.4 ± 1.8	7.8 ± 2.2	<0.001
ACS	18.3 ± 3.1	23.9 ± 3.6	
Small vessels (≤2.75 mm)	10.5 ± 2.2	14.0 ± 2.6	0.73
Large vessels (>3.0 mm)	13.3 ± 3.3	15.8 ± 3.7	
**TLR rate (%)**	5.6 ± 1.3	6.0 ± 1.3	
Stable CAD	3.0 ± 1.3	3.7 ± 1.5	0.049
ACS	8.5 ± 2.3	8.5 ± 2.3	
Small vessels (≤2.75 mm)	3.6 ± 1.3	4.2 ± 1.4	0.12
Large vessels (>3.0 mm)	8.7 ± 2.8	8.7 ± 2.8	

MACE, major adverse cardiovascular event comprising of cardiovascular death, non-fatal myocardial infarction, and target lesion revascularization (TLR).

*p-value has been calculated between stable CAD vs. ACS and small vs. large vessels at 12 months.

### Reasons leading to target lesion revascularization after drug-coated balloon-only strategy

In 14 cases (74%) of TLR, acute recoil ≥30% after predilatation was found to be the probable reason for TLR. In contrast, dissections leading to reduced blood flow were not found as a reason for TLR. Two TLR events occurred in lesions that were bailout stented (11%). Only in three cases (16% of TLR events and 1.2% of the whole population) there was no evident reason for restenosis after the DCB-only strategy (Table 5).

**TABLE 5:** Reasons for target lesion revascularization (TLR) after DCB (drug-coated balloon)-only percutaneous coronary intervention.

**TLR events (12 months)**	19	4.7
Dissection with <TIMI3 flow after DCB	0	0
Acute recoil >30% accepted after DCB	14	74
TLR in BOS	2	11
No specific reason	3	16

DCB, drug-coated balloon; BOS, bailout stenting; TLR, target lesion revascularization.

## Discussion

The DCB-only strategy in the treatment of *de novo* coronary lesions is increasing worldwide in a variety of clinical and anatomical scenarios (17). However, so far RCTs with clinical endpoints have tested this strategy only in small vessels, HBR patients, and in patients with non-ST elevation myocardial infarction (7, 8, 11). RCTs with surrogate endpoints such as late-lumen loss or post-PCI fractional-flow reserve have been conducted in the treatment of bifurcation lesions and patients with ST-elevation myocardial infarction (19, 20). In addition to RCTs, there is still a need for real-world studies of the DCB-only strategy in every day interventional management of CAD patients. In this study, the efficacy of the DCB-only strategy was separately assessed in subgroups such as in stable CAD, in ACS, as well as in small and large vessels. The reasons for the technical failure of the DCB-only strategy as well as the underlying causes of TLR after DCB-only PCI were also studied.

DCBs differ significantly in their excipient technology and drug concentration, and therefore results obtained with one DCB cannot be extrapolated to other DCBs. Here, we studied the clinical performance of a DCB with CEE as an excipient and containing less paclitaxel (2 μg/mm^2^) than the majority of other DCBs in the market in an all-comers population needing revascularization. The theoretical advantage of a smaller concentration of paclitaxel is the lesser risk of potential cytotoxic side effects at the target lesion. On the other hand, it may also result in a suboptimal drug concentration in challenging anatomical subsets, such as severely calcified vessels. Rotational atherectomy was used in 11% of cases before the DCB-only strategy demonstrating that also complex calcified lesions were treated by this approach. Ischemia-driven TLR rate has ranged between 0 and 6% at 9–12 months in previous studies of paclitaxel-coated DCBs (4–6, 8–10). In the current study, the TLR rate was found similar (5.6%) at 12 months. Importantly, the TLR rate was only 1.2% in lesions that fulfilled the international consensus document guideline criteria after the DCB application (17). This finding strongly underlines the importance of proper lesion preparation in the DCB-only strategy and the fact that bailout stenting should be used in case of technical failure.

The clinical efficacy of the DCB using CEE as an excipient has been demonstrated in an RCT for the treatment of ISR of DES, where it was found non-inferior compared to another DCB coated with a higher concentration of paclitaxel and iopromide as an excipient (3). Our study is the first that specifically addresses the efficacy of this DCB device for the treatment of *de novo* coronary artery lesions. Previously, one registry study has been published using the same DCB in an all-comers population (21). However, in that study, only 63% of the lesions were *de novo* and most of them were located in small coronary arteries. Furthermore, that study did not address the efficacy of the DCB-only strategy as the hybrid DES + DCB PCI was allowed (44% of cases) in very long lesions and bifurcations (DES in the main branch and DCB in the side branch). Finally, the vast majority of patients in that study had stable CAD (83%), which contributes to the lower TLR (2.2% in the *de novo* group) and MACE (3.5% in the *de novo* group) rates in comparison to our study.

The performance of the DCB-only strategy is important to be validated in different clinical and anatomical scenarios. Here, we studied subgroups such as large and small vessels as well as stable CAD and ACS. We found that the clinical efficacy of the DCB with CEE as an excipient was comparable regardless of the vessel size with no significant difference in the rates of MACE or TLR. As expected, we found that MACE, TLR, and cardiovascular mortality were substantially higher in the ACS group as compared to the stable CAD group after the DCB-only treatment. The TLR rate was only 3.0% in patients with stable CAD but more than double in the ACS population (8.5%) at 12 months. Similarly, the MACE rate was low in the stable CAD group (5.4%) but over triple in the ACS population (18.3%) at 12 months. Noteworthy, 12% of ACS patients had ST-elevation myocardial infarction. This all-comers study included elderly patients with comorbidities (e.g., 24% were over 80 years old, 34% had baseline anemia, 22% had had a prior myocardial, and 9% had a prior stroke), which also contributed to the relatively high MACE rate in the ACS population.

The overall primary success rate of PCI using the paclitaxel DCB with CEE as an excipient was 83% in *de novo* lesions. The most important reason for technical failure was the need for bailout stenting. Eventually, the bailout stenting was performed in 9% of DCB PCIs, whereas it was omitted in 40% of cases that did not fulfill the international consensus group recommendation. Bailout stenting was at the operator’s discretion. Probably one important reason for deferring bailout stenting and accepting suboptimal DCB-only results was HBR. The rate of bailout stenting is in line with previously published studies (4–6, 9, 10). To better understand the reasons for TLR, we analyzed the angiographical results after DCB-only PCI. In 75% of cases, acute recoil was more than 30% after DCB-only PCI, and this result had been accecpted without bail-out stenting. This finding is important in respect of the potential causes of the failure of this strategy. In contrast, major dissections (causing ≤TIMI3 flow) were not found in cases of TLR. Only in three cases of TLR, we did not find a specific reason for restenosis. The treatment of *de novo* coronary lesions with this DCB was safe as no acute vessel occlusion occurred. Also, previous studies have demonstrated a very low risk of acute vessel closure after the DCB-only strategy (typically from 0 to 0.2%) (7–10, 22).

Bleeding after PCI is becoming a major clinical concern as it increases mortality by 7-fold a year (16). The DCB-only strategy together with short DAPT is appealing to the elderly, anticoagulated, or frail patients who are prone to bleeding complications during DAPT (8). In this study, over half of the patients had at least one risk factor for bleeding and 22% were on OAC. The recommended duration of DAPT after DCB-only PCI in stable CAD is currently 1 month (17). However, the recommendation of DAPT duration by the manufacturer of this DCB was three months in stable CAD at the time of the study. The mean duration of DAPT was even longer than that, i.e., 3.8 ± 3.6 months in stable CAD, probably because 27% of patients also received DES in another lesion in the index PCI. In addition to short DAPT, even SAPT is possible after DCB-only PCI in patients at extreme HBR such as in patients that have suffered recent life-threatening bleeding or require upcoming urgent non-cardiac surgery. Furthermore, in case of severe bleeding, the whole antithrombotic treatment can be ceased as no metallic implant is placed in the coronary artery. In our study, 13 and 15% of patients having stable CAD and ACS were discharged with SAPT, respectively. Moreover, in 4% of PCIs perioperative ADP receptor blocker was omitted. In the ACS cohort, the mean duration of DAPT was 7.2 ± 6.7 months. The DAPT duration in the ACS population was shorter than that recommended by the current guidelines (12 months), which also reflects the HBR characteristics of the study population and the need for individual tailoring of antiplatelet therapy. Despite shorter DAPT than recommended by the guidelines, the incidence of BARC types 2–5 bleedings was as high as 24 and 32% in patients with stable CAD and ACS, respectively, by 12 months.

The most important limitation of this study is that it is a retrospective single-center registry study. Second, there is a selection bias regarding patients that were treated by DCB-only PCI instead of stenting with DES and therefore HBR patients are over-presented in this cohort. It is not known how much primary DES implantation in multivessel PCI contributed to MACE regarding myocardial infarctions and cardiac deaths. Furthermore, it is not known in how many cases significant acute recoil or flow-limiting dissection after predilatation led to primary DES implantation. Moreover, there are no matched controls in this study such as patients receiving another DCB or DES.

## Conclusion

In conclusion, the DCB-only strategy with the paclitaxel DCB with CEE as an excipient is both safe and effective for the treatment of *de novo* lesions in small and large coronary arteries in an all-comers population. Careful lesion preparation according to the recommendations of DCB-only PCI guidelines, especially not allowing significant recoil, results in a low rate of restenosis after the DCB-only approach.

## Data availability statement

Data are not available given the further studies that the authors are planning on the dataset. Requests to access the datasets should be directed to TR, tuomas.rissanen@siunsote.fi.

## Ethics statement

The trial was approved by the Research Ethics Committee of the Northern Savo Hospital District. Written informed consent for participation was not required for this study in accordance with the national legislation and the institutional requirements.

## Author contributions

All authors contributed to the collection of the data, interpretation and writing the original manuscript.
